# Supplementary material for: Are carbon emissions trading and green financial instruments synergistic? -Comprehensive quantitative research based on content analysis
Source: PLoS One. 2024 Mar 7;19(3):e0298601. doi: 10.1371/journal.pone.0298601 (PMC10919601; doi:10.1371/journal.pone.0298601)
Supplement: S1 Data — (ZIP) [file pone.0298601.s001.zip › Data/地级市Z_F工作报告.zip!/╜¡╦╒/╦╒╓▌╩╨/└┤╫╘╣1⁄2╓┌║┼í╛┬φ┐╦╩2╛▌═°í┐_2011.docx]

各位代表：

　　现在，我代表苏州市人民政府，向大会报告工作，请予审议，并请市政协各位委员和其他列席人员提出意见。

　　2011年工作回顾

　　过去一年里，市政府在中共苏州市委的正确领导下，紧紧围绕建设“三区三城”和率先基本实现现代化的目标，团结依靠全市人民，坚持不懈稳增长、调结构、抓创新，持之以恒重统筹、惠民生、促和谐，较好地完成了市十四届人大四次会议确定的目标任务，推动了经济社会又好又快发展。

　　转型升级取得成效。经济持续平稳增长，运行质量进一步提高。全市地区生产总值突破1万亿元，预计达到10500亿元，按可比价计算比上年增长12%；地方一般预算收入1100.9亿元，增长22.2%；全社会固定资产投资4502亿元，增长24.4%。实现工业总产值3.3万亿元，增长17%；其中规模以上工业产值2.8万亿元，增长17.7%。新兴产业产值增长24.7%，占规模以上工业产值的比重达到38.1%，比上年提高9.3个百分点。工业化与信息化深入融合，涌现出一批“两化”融合示范区、示范企业、示范项目。传统产业改造力度加大，技术改造投资占工业投资的比重达到68.1%。新增制造业地标型企业7家，累计达到21家。服务业规模持续扩大，实现增加值4470亿元，占地区生产总值的比重提高到42.6%。省、市级服务业集聚区达到60个。新增制造业分离发展现代服务业企业466家，累计已达738家。加强城市商圈和特色商业街建设，实施“万村千乡”市场工程和便利消费进社区、便民服务进家庭工程，城乡市场繁荣活跃，实现社会消费品零售总额2830亿元，增长17.8%。我市被确定为国家电子商务示范城市，苏州高新区馨泰社区被评为全国社区商业示范社区。旅游业保持较快发展，接待入境游客296万人次、国内游客7775万人次，分别增长12%和13%；旅游总收入达到1190亿元，增长17%。我市成为全国旅游标准化示范城市和全国首批智慧旅游试点城市。文化产业实现增加值480亿元，增长25%。阳澄湖数字文化创意产业园成为国家级数字出版基地。年末金融机构人民币存贷款余额分别比年初增长11.6%和12.6%。全年保费收入增长15.8%。全社会融资总量再攀新高，新增融资超过3500亿元。农业现代化加快推进，综合生产能力不断增强。新建高标准农田6.9千公顷，建设粮油万亩高产示范片29个，粮食生产喜获丰收。新增高效农业面积8.5千公顷、设施农业面积4千公顷，现代农业园区扩大到40千公顷，太仓现代农业园区被评为国家农业产业化示范基地。新添无公害农产品、绿色食品、有机食品189个。吴江市成为国家级农业标准化示范县（市）。发展农产品加工和配套服务业，新增国家级农业产业化龙头企业2家，全市农业产业化龙头企业销售收入达到1650亿元，增长14%。开放型经济向更高层次迈进，经济国际化水平又有新提升。进出口总额突破3000亿美元，增长9.5%。一般贸易出口所占比重达到23.7%，比上年提高3.5个百分点。利用外资结构持续优化，实际利用外资89.1亿美元，增长4.4%。其中服务业实际利用外资29.3亿美元，新兴产业和高新技术项目实际利用外资33.5亿美元；新增外资企业地区总部和功能性机构45个。与港澳台的合作交流继续深化，在新兴产业、现代服务业、科技文化等领域取得新的进展。服务外包发展迅速，实现接包合同额35.4亿美元、离岸执行额20.1亿美元，分别增长56%和57.9%。江苏花桥国际商务城成为全国首个服务外包认证国家示范区。“走出去”步伐加快，中方境外协议投资额达到7亿美元，增长48%，总额位居全省第一。开发区“二次创业”成效明显，新增张家港经济技术开发区、太仓港经济技术开发区2个国家级开发区。南北挂钩合作更加紧密，苏州宿迁工业园区在全省共建园区考核评比中获得第一名，苏州南通科技产业园、苏州盐城沿海合作开发园区加快建设。加强对口援建，新疆霍尔果斯特殊经济开发区大型综合体苏新中心、东部产业转移园、产业园集宿区等项目全面启动。

　　创新力度不断加大。创建国家创新型城市试点工作积极开展，我市及各县级市、区全部进入全国科技进步先进行列。高新技术产业产值增长17.6%，占规模以上工业产值的比重达到37.3%，比上年提高0.7个百分点。4个项目获得国家科学技术奖，其中有机发光显示项目获得国家技术发明一等奖。加大研发投入力度，全社会研究与试验发展经费支出占地区生产总值的比重达到2.45%，比上年提高0.11个百分点。促进科技与金融结合，增加科技型企业信贷风险准备金，发放“科贷通”专项贷款21.7亿元。我市与中国科学院、北京大学、清华大学的战略合作深入推进。国家“千人计划”创业投资中心挂牌运作，新增国家认定企业技术中心5家以及省级工程技术研究中心、重点实验室、企业技术中心74家，我市获批建设核电领域首个国家级工程技术研究中心。中科院苏州纳米技术与纳米仿生研究所二期、中科院苏州生物医学工程技术研究所二期和浙江大学苏州工业技术研究院等项目加快建设，苏州国科数据中心一期建成使用，吴中区中科院医药研发中心揭牌成立，苏州工业园区国家纳米高新技术产业化基地获得批准，苏州高新区医疗器械产业集群列入国家创新型产业集群建设工程。认真实施姑苏人才计划和“1010”工程，精心组织苏州国际精英创业周活动，在海外举办“赢在苏州”国际精英系列创新创业大赛，聚才环境更趋优化。我市新增昆山国家高新技术产业开发区、苏州大学2个国家海外高层次人才创新创业基地；新增入选国家“千人计划”人才20名，累计达到50名，居全国地级市首位；新增省高层次创新创业人才67名，累计达到205名，连续五年保持全省第一。知识产权创造充满活力，专利申请量、授权量均列全国大中城市第一位。国家专利审查协作江苏中心落户苏州高新区。落实技术标准战略和名牌发展战略，新增2个国际标准化组织和2个国家标准化组织，苏州工业园区成为全国首个国家纳米技术产业化标准化示范区，吴江市国家通信光电缆产品质量监督检验中心通过验收。国家商标战略实施示范城市建设积极推进，新增中国驰名商标14件，累计达到58件。创新体制机制，经济发展活力不断增强。健全国有企业领导干部绩效考评机制，注重公司监事会建设，国有资产监督管理得到加强。改进政府投入方式，探索采取奖励、担保、设立投资基金等形式扶持产业发展。创新金融发展，成立了苏州产权交易中心和市信用再担保公司，东吴人寿保险公司获准筹建，地方金融业态更加丰富。国家开发银行在地级市设立的首家分行落户苏州。新增东吴证券等上市公司18家,募集资金214亿元，又有8家企业获批发行企业债券89亿元。积极发展小额贷款公司，累计开业72家，注册资本达到193亿元，累计发放贷款超过1000亿元，有效缓解了中小企业融资难问题。促进民营经济快速健康发展，私营企业和个体工商户净增注册资本1394亿元，上缴税收612亿元，分别增长19.6%和15.8%。

　　城乡建设统筹推进。新一轮城市总体规划调整上报工作顺利完成，滨湖新城、高铁新城规划和中环快速路规划抓紧编制，沿独墅湖地区、虎丘周边地区等区域的城市设计积极开展。京沪高速铁路苏州段及配套场站建成投运，苏州火车站北广场按期完工，南站房和南广场配套工程加快推进。太仓疏港高速公路抓紧建设，苏虞张公路快速化改造启动实施，230省道吴江北段完成改造任务。高铁快速路建成通车，东环快速路南延、南环快速路西延二期等工程如期开工。轨道交通1号线进入空载试运行阶段，2号线建设进展顺利。干将路综合整治基本完成，一批城市道路修建竣工。苏州港新增万吨级以上码头泊位2个，货物吞吐量达到3.8亿吨，集装箱吞吐量超过450万标箱，其中太仓港区货物吞吐量突破1亿吨，集装箱吞吐量超过300万标箱。张家港复线船闸工程和苏南运河、杨林塘航道升级工程加紧实施。加强电网建设，投产110千伏及以上线路806公里。新增人防设施75万平方米。市容环卫保洁水平不断提高，城市道路交通和车辆管理得到强化。餐厨垃圾收集处置二期工程加快建设，生活垃圾焚烧发电三期工程开工兴建，七子山垃圾填埋场渗沥液处理站提标改造工程全面建成，新设了一批建筑垃圾临时归集点。城乡一体化发展综合配套改革深入开展，在城乡规划有机融合、农村土地使用制度改革、社会保障制度并轨、金融服务创新等方面取得实质性进展，我市成为全国农村改革试验区。优化城乡布局和资源配置，完成镇村布局规划修编工作，确定农村保留村庄和集中居住点，全市农村88%的工业企业进入工业园，43%的农民实现集中居住，80%的承包耕地实现规模经营。开工建设农民安置房1800万平方米，全年有5万农户进城进镇落户。新增社区股份合作、土地股份合作和农民专业合作组织354家，累计达到3397家，持股农户占农户总数的比重提高到95%。集体经济发展壮大，村均年集体收入达到503万元，增长12%，涌现出一批规模大、竞争力强、管理模式新的集体经济组织。农村金融服务不断改善，农业保险体系加快构建，农业担保业务继续扩大。加大强农惠农力度，全年财政农林水事务支出达到77.8亿元，增长22.7%。注重农村社区建设，管理服务水平明显提升。

　　社会事业繁荣进步。坚持教育优先，加快教育现代化步伐。学前教育健康发展，新增省优质幼儿园18所，累计达到270所。省义务教育优质均衡改革发展示范区建设取得实效，义务教育办学水平整体提升。外来人口子女在公办学校接受义务教育的比例超过70%。推动普通高中多元化、特色化发展，苏州中学成为国家教育体制改革创新人才培养试点学校。职业教育办学活力不断增强，我市被列为首批省职业教育创新发展实验区，2所高等职业技术学院分别成为国家示范和骨干高等职业技术院校。市立达中学新校、景范中学金阊校区建成启用，盲聋学校新校基本建成。支持在苏高校改革发展，6所高校成为国家教育体制改革项目试点学校。完善公共文化设施，苏州美术馆新馆、文化馆新馆和名人馆落成启用，一批县级市（区）标志性文化工程如期建成。镇（街道）文化站、村（社区）文化活动室达标率分别超过90%和95%，送书、送戏、送电影和“群星璀璨·2011”等文化惠民活动积极开展，我市成为国家首批公共文化服务体系示范区创建市。有线数字电视高清、双向升级有序实施。苏州日报报业集团、苏州广播电视总台所属经营性事业单位完成转企改制，组建了苏州报业和苏州广电两大传媒集团公司。虎丘地区综合改造加速推进，桃花坞历史文化片区综合整治保护利用、古城墙保护性修复和古建老宅保护修缮等工程顺利开工。吴文化考古研究获得重大成果，木渎春秋城址入选全国十大考古新发现。滑稽戏《顾家姆妈》被评为国家舞台艺术精品剧目和第十二届中国戏剧节优秀剧目奖第一名，并获中国戏曲学会奖，我市表演艺术家获得中国戏剧梅花大奖。吴江市戏曲文化生态保护区建设入选国家文化创新工程重点项目，区域文化联动项目通过国家验收。太湖文化论坛首届年会成功举办。中国工艺文化城首期建成开业。哲学社会科学研究和普及工作深入开展。第二轮《苏州市志》进入总纂阶段。市档案馆、苏州工业园区档案馆通过国家一级档案馆测评。城乡医疗卫生服务体系全面建成，国家基本药物制度进一步落实，基层医疗卫生机构综合改革加快实施。苏州大学附属第一医院主体迁建一期工程启动建设，苏州大学附属儿童医院园区总院、市立医院本部的门诊急诊楼等项目按计划推进。医疗便民服务“一卡通”积极推广，重性精神病患者免费治疗工作稳步开展。金阊区留园街道社区卫生服务中心成为全国示范单位。急救体系逐步完善，急救能力明显增强。国家卫生城市、国家卫生镇通过复审。幸福家庭世代服务工程全面启动，流动人口计划生育服务和管理得到加强，0～3岁科学育儿项目有效实施，我市成为全国幸福家庭创建试点市和全国青少年健康人格工程试点市。顺利通过实施妇女儿童“十一五”规划终期评估验收，妇女儿童事业又获可喜成果。广泛开展全民健身服务，经常参与锻炼的人数不断增加。市区五卅路体育场馆改造和市体育运动学校配套工程抓紧建设。健全少数民族服务保障体系，组织开展“和谐寺观教堂”创建活动，民族团结、宗教和睦的氛围继续巩固。我市创建全国双拥模范城实现“五连冠”，国防教育、国防动员工作成效显著。国际交流与合作更加密切，新增国际友好城市1个，累计达到42个，我市再次获得国际友好城市交流合作奖。侨务工作取得新成绩，张家港市、金阊区白洋湾街道分别被国务院侨务办公室评为海外人才中国创业示范基地和全国社区侨务工作明星社区。

　　生态环境继续改善。切实抓好东太湖、阳澄湖和金墅港水源地综合整治，强化蓝藻防治，加快同城第二水源地建设，集中式饮用水源水质达标率保持100%。深入开展太湖流域水环境整治，实施走马塘延伸拓浚和农村河道疏浚畅流工程，下大力气治理黑臭河道，城乡水环境有所改善。加强城乡污水处理，城镇生活污水处理率达到92%，太湖、阳澄湖保护区农村生活污水处理率分别达到78%和72%，其他地区生活污水处理率达到51%。继续实施“蓝天工程”，切实治理扬尘污染，整治机动车尾气超标排放，坚持黄标车区域限行，推行油气回收综合利用，完善灰霾天气预报预警机制，建立灰霾监测数据综合显示平台，努力改善空气质量状况。开展重点区域、重点行业和重点企业的重金属污染整治，规范辐射许可和危险废物转移。认真实施污染减排计划，加快淘汰落后产能，关闭化工企业125家。落实最严格的耕地保护和占补平衡制度，通过土地复垦整理新增耕地687公顷，土地节约集约利用水平逐步提高。我市在全国率先建成国家节水型城市群。再生资源（甪直）产业园奠基开工，再生资源回收网点增加到277个。注重资源综合利用，积极发展循环经济，苏州城市循环经济发展共性技术开发与应用研究项目通过国家验收，我市成为国家餐厨废弃物资源化利用和无害化处理首批试点城市。积极推行清洁生产，完成214家重点企业的清洁生产审核工作。开展争创“能效之星”活动，企业节能意识普遍增强。大力加强生态建设，认真落实生态补偿政策。实施环古城风貌保护三期、石湖景区建设等重大景观绿化工程，市区新增绿地505万平方米。推进农村河湖农田林网构建、绿色通道提升、生态片林营建等工程，整治复绿山体宕口，农村新增林地绿地6547公顷，陆地森林覆盖率达到25%。国家级野生植物原生境保护点启动建设，苏州太湖国家湿地公园入选首批国家级湿地公园。我市实现全国环境优美镇创建“满堂红”，吴江市和吴中、相城区建成国家生态市（区），我市创建国家生态市以及市区、张家港、常熟、昆山市创建国家生态园林城市通过考核验收。苏州工业园区、苏州高新区成为首批国家生态工业示范园区。吴江、常熟市先后获得中国人居环境奖。

　　民生质量逐步提高。大力实施富民工程，城镇居民人均可支配收入达到33070元，增长13.2%；农民人均纯收入达到17040元，增长16.3%。市十四届人大四次会议确定的实事项目全面完成年度任务。实施积极的促进就业政策，全市新增就业17.3万人，城镇登记失业率为2.72%，苏州籍高校毕业生就业率达到95.7%，零就业家庭实现动态清零。免费培训城乡劳动者38.6万人。加强社会保障体系建设，城镇职工养老、医疗、失业、工伤、生育五大社会保险参保覆盖面和社保基金征缴率均保持在99%以上。被征地人员保养金待遇从每月430元提高到每月510元。城乡居民最低生活保障标准统一提高到每月500元，在全国地级市中率先实现城乡低保全市同一标准和全面并轨。开展扶贫帮困活动，累计发放各类社会救助资金17.8亿元，惠及困难群众17.1万人次。失业保险金在领人员开始享受职工医疗保险待遇，在全国率先实施了针对低收入家庭的专项医疗救助。红十字救助、慈善募捐等活动积极开展。构建残疾人社会保障和服务体系，向低保家庭中的重度残疾人每月发放80元补贴金。我市被评为全国残疾人工作示范城市。加强养老服务和管理，新增养老床位5400张，尊老金发放范围由90周岁以上的老年人扩大至80周岁以上的老年人。出台惠民殡葬政策，免除城乡居民殡葬基本服务费用。加快建设保障性安居工程，新建公共租赁住房24549套（间）、经济适用住房2381套、限价商品住房12393套，新增廉租住房501套，解危修缮危旧住房235万平方米，南环新村危旧房解危改造工程扎实推进。市区完成123万平方米老住宅小区、14万平方米零星居民楼综合整治任务，实施城区居民家庭“改厕”7942户，改造城中村（无地队）22个，实行天然气置换3.3万户。新增参加住房公积金制度的职工52万人，职工使用住房公积金116亿元。市区新辟公交线路12条，新增公交车700辆，新投放出租车400辆，乘坐公交车实行刷IC卡6折优惠和空调车季节性优惠，并对义务教育阶段学生和残疾人予以免费。新建改建城乡农贸市场89个，肉类蔬菜流通追溯体系初步建立。设立市食品质量安全监控中心，开展食品非法添加和滥用食品添加剂专项整治，食品、药品、保健品、化妆品、医疗器械安全监管得到加强。依法打击侵犯知识产权和制售假冒伪劣商品行为，有效改善了消费环境。粮食批发市场功能更趋完善，粮食储备规模进一步扩大。加强价格调控监管，落实保供稳价安民措施，建设农产品平价直销网点，启用重要民生商品价格采集系统，居民消费价格总水平涨幅逐步得到控制。

　　社会管理全面加强。坚持以社会主义核心价值体系巩固共同思想基础，广泛开展精神文明创建活动，市民文明素质和社会文明程度不断提高，1人荣获全国诚实守信道德模范称号，46人荣登“中国好人榜”，25个单位、村镇被中央文明委评为全国文明单位、文明村镇。公共文明意识普遍增强，社会志愿服务蔚然成风。深化文明城市长效建设，苏州市、张家港市蝉联全国文明城市。全民科学素质又有较大提高，我市再次实现全国科普示范县（市、区）创建“满堂红”。推进基层民主法治建设，法治城市创建工作取得新进展。法制教育宣传扎实有效，常熟、昆山市和平江区分别被评为全国法制宣传教育先进县（市）、先进单位。开展法律进社区、进乡村活动，增设了一批法律服务窗口。加强法律援助，全市法律援助中心共受理援助案件7136件，增长45.6%。镇（街道）、村（社区）和企业人民调解组织逐步完善。社区矫正工作力度加大。和谐社区、文明社区建设不断加强，城乡社区自治水平持续提升。深入开展平安苏州创建活动，创新社会管理综合治理，深化“创意警务”品牌建设，促进社会和谐稳定。严厉打击违法犯罪行为，着力维护人民生命财产安全。严密防范境内外敌对势力的渗透破坏活动，切实保障国家安全。改进外来人口管理，实施流动人口居住证制度。制定应急管理规划，市、区应急管理工作机构和网络全面建立，市应急指挥平台建成运行，应急综合演练走向常规化、制度化。落实信访工作长效机制，积极化解信访积案，努力保障群众的合法权益。强化安全生产责任，深入开展安全事故隐患排查治理和“打非治违”专项行动，杜绝了重特大事故发生。

　　政府自身建设进一步强化。认真做好政府法制工作，全年提请市人大常委会审议通过地方性法规3件，颁布政府规章9件，制定政府规范性文件18件。抓好相对集中行使行政复议权试点工作，积极化解行政争议。深化行政审批制度改革，规范审批程序，创新审批方式，完成第五批行政审批事项清理规范工作，减少行政审批事项79项。经济发达镇行政管理体制改革试点稳步开展。认真贯彻落实党风廉政建设责任制，着力打造廉洁政府。秉持“机关就是服务、公务员就是服务员”的理念，加强机关作风效能建设，实现行政权力网上公开透明运行全覆盖。注重公务员教育，创新培训形式，组织开展了市级机关科职公务员自主选学培训。完善行政服务中心、便民服务中心各项制度，工作作风不断改进，工作效能稳步提高。加强公务接待管理，严格执行公务用车购置和使用规定，清理和规范庆典、研讨会、论坛等活动，切实控制和减少各类会议。因公出国（境）人数在前三年压缩20%的基础上又有明显下降。深化财政预算执行审计、绩效审计和经济责任审计，政府资金使用效益进一步提高。强化统计监测预警，服务功能得到更好发挥。市政府自觉接受市人民代表大会及其常务委员会的法律监督、工作监督和市政协的民主监督，坚持重大事项向市人大常委会报告、向市政协通报，注重发挥各民主党派、工商联、无党派人士和人民团体的参政议政作用，认真实施关于“加强扬尘污染防治管理”和“加快发展社会养老服务业”的议案，办复人大代表建议217件、政协提案510件。

　　各位代表，经过全市人民的不懈努力，苏州在率先发展、科学发展、和谐发展的道路上迈出了坚实的步伐，取得了“十二五”开局之年的全面胜利。在此，我代表市政府向全市人民，向人大代表、政协委员，向离退休老同志，向各民主党派、工商联和无党派人士，向各人民团体，向驻苏解放军、武警和消防官兵，向国家和省驻苏单位，向参与、支持和关心苏州现代化建设的海内外朋友，表示衷心的感谢和崇高的敬意！

　　回顾过去的一年，我们也看到，经济社会发展还存在不少问题，政府工作与人民期待相比仍有差距，主要是：产业结构有待优化，自主创新能力亟待增强，高层次人才仍然不足，资源环境约束加剧，外贸形势不容乐观，部分企业生产经营困难、效益下降；物价上涨较快，城乡居民收入水平仍需不断提高；社会事业发展相对滞后，经济社会转型中的矛盾持续显现，社会建设与管理任务加重；政府行政效能和服务水平需要进一步提升。对此，我们将采取有效措施，努力加以解决。

　　2012年主要任务

　　今年是实施“十二五”规划承上启下的重要一年，是我市率先基本实现现代化的突破之年。总体来看，我们面临的形势严峻复杂，但仍然处于大有可为的重要战略机遇期。我们将主动适应宏观环境的新变化，深刻认识苏州发展阶段的新特征，牢牢把握人民群众的新期盼，认真落实中央和省委、省政府对我市发展的最新要求，切实增强忧患意识，始终坚定必胜信心，扬长避短，趋利避害，在克难求进中应对挑战，在开拓创新中破解难题，在转型升级中增创优势。根据中共苏州市委十一届二次全体（扩大）会议精神，今年政府工作的总体要求是：以邓小平理论和“三个代表”重要思想为指导，深入贯彻落实科学发展观，紧扣率先基本实现现代化核心任务，坚持稳中求进、又好又快工作导向，突出加快转型、稳定外贸、兴盛文化、广惠民生等重点，深入实施创新引领、开放提升、城乡一体、人才强市、民生优先、可持续发展战略，确保经济社会平稳较快发展，奋力开创“三区三城”建设新局面。

　　全市经济社会发展的主要预期目标是：地区生产总值增长12%左右，地方一般预算收入增长13%左右，服务业增加值占地区生产总值的比重提高1.5个百分点，新兴产业产值占规模以上工业产值的比重提高5个百分点左右，全社会固定资产投资增长15%，社会消费品零售总额增长17%，进出口总额增长5%，实际利用外资与上年持平，城镇居民人均可支配收入和农民人均纯收入增长13%左右，居民消费价格总水平涨幅控制在4%左右，城镇登记失业率控制在4%以内，全社会研究与试验发展经费支出占地区生产总值的比重达到2.6%，单位地区生产总值能源消耗下降、主要污染物排放总量削减完成省下达的任务。

　　新的一年里，我们将重点在以下十个方面下功夫、抓落实。

　　一、着力加快转型发展

　　调整产业结构，提高产业层次。加快发展物流、金融、商务服务、科技和信息服务等生产性服务业，提升发展各类消费性服务业。新建服务业集聚区10个左右，努力实现龙头带动，形成规模优势。推动制造业企业分离发展现代服务业，鼓励支持科技研发、物流配送、贸易营销、设计策划等分离外包，新增一批销售规模超过50亿元的服务业企业。建设战略性新兴产业基地，壮大特色产业集群，引导建立产业联盟。培育掌握核心技术、具有自主知识产权和较强国际竞争力的地标型企业。实施“万企升级”计划，以高新技术和先进适用技术改造传统产业，增强市场竞争力。促进信息技术在工业领域的应用，提高工业化、信息化融合水平。优化产业布局，加快建设苏州工业园区—相城区合作经济开发区和各类产业园区；加强跨江联动开发，实施工业产业转移规划，为新兴产业和现代服务业腾出更多发展空间。

　　继续扩大内需，拉动经济增长。加大民生事业、基础设施的投入力度，发挥政府投资的带动作用，引导企业资金投向新兴产业和现代服务业。集中力量建设总投资7900亿元的230项重大项目，年内完成投资超过1600亿元。加强项目储备，增强投资后劲，实现持续发展。完善城乡商贸流通体系，发展社区商业、农村商业。重点打造以观前、石路、南门为代表的古城区商业板块和环金鸡湖商贸区、狮山商贸区，大力发展电子商务、会展等新兴商贸业态。优化消费环境，培育消费热点。深入实施旅游业全面提升计划，提高旅游标准化水平，改进旅游服务，扩大旅游消费。着力形成古城、太湖、中部和沿江四大旅游集聚区，推进旅游度假区建设，加快5A级景区创建步伐，全面启动智慧旅游工程。拓展大众文化消费市场，开发特色文化消费，增加文化消费总量，提高文化消费水平。

　　二、着力推动自主创新

　　围绕创建国家创新型城市的要求，坚持依靠创新赢得先机、赢得优势、赢得未来。强化资源与资本、科技与产业、产品与市场的结合，培育一批创新先锋企业，突破一批产业关键技术，加速转化一批重大创新成果。实施高新技术产业倍增工程，力争产值增长20%以上，新增高新技术企业200家。完善“科技镇长团”、“企业科技行”等产学研合作方式，推动高校、地方、企业共建产业技术研究院。促进科技与金融紧密结合，加快推进国家“千人计划”创业投资中心和沙湖股权投资中心二期建设。加大对研发活动的支持力度，努力形成以政府投入为引导、企业投入为主体、社会资本广泛参与的多元化科技创新投入体系，以高强度投入支撑高水平创新。全力打造独墅湖科教创新区、苏州科技城、昆山阳澄湖科技园等创新核心区。积极构建开放共享的技术研发平台、检验检测平台、科技信息平台和技术转移平台，支持设立工程技术研究中心和企业技术中心。大力引进先进技术、先进装备和先进管理方法，吸引外资企业来苏设立研发机构。建设苏州自主创新服务广场，努力提供优质高效的科技服务。健全知识产权保护长效机制，确保通过全国版权示范城市创建验收，建好国家专利审查协作江苏中心。做好质量强市工作，争创国家级省级优质产品生产示范区、知名品牌示范区和中国驰名商标，扩大品牌效应，提高标准化水平。深入实施“姑苏人才计划”和“1010工程”，加快柔性引进海外智力，积极承办国家“千人计划”太湖峰会，继续办好苏州国际精英创业周和“赢在苏州”创新创业大赛，持续发挥人才工作优质品牌效应，大力集聚高层次高技能人才和高水平创新创业团队。

　　三、着力深化改革开放

　　加大重点领域和关键环节的改革力度，持续增强发展动力和活力。加强国有企业监管，强化绩效考评和收益分配管理。优化国有资源配置，提高国有资产运行质量和效益。推进行政审批制度改革，探索建立相对集中行政许可权制度，继续削减行政审批事项，对审批项目开展动态评估和效果评估。做好经济发达镇行政管理体制改革试点工作。深化医药卫生体制改革，重点抓好公立医院改革，促进基层医疗卫生机构健康运行。在完成事业单位清理规范工作的基础上，实施事业单位分类改革。规范发展行业协会和中介机构，扶持培育公益性社会组织。积极争取国家增值税制度改革试点，力争苏州工业园区、苏州高新区、江苏花桥国际商务城进入国家现代服务业综合试点行列。重点开展面向科技进步、中小企业发展和城乡一体化等方面的金融创新，引导和支持企业通过公司上市、发行债券、中期票据、信托和融资租赁等多种渠道，不断扩大融资规模。做大做强地方法人金融机构，东吴人寿保险公司实现挂牌开业。发展创业投资基金、私募股权基金，争取我市成为全国股权投资集聚区试点城市。改进中小企业金融服务，增加对中小企业的信贷支持。引导保险公司开展科技保险、文化产业保险、中小企业贷款保证保险。培育龙头型、骨干型民营企业和高成长型中小企业，促进民营经济转型升级。更加积极主动地实施对外开放，巩固扩大开放型经济的领先优势。千方百计保市场、保订单，引导和鼓励企业开发新产品、拓展新市场。以建设全国加工贸易转型升级试点城市为契机，推进加工贸易向高端制造、营运管理、研发设计、采购销售等高附加值领域延伸。抓好出口基地和出口品牌建设，提高一般贸易竞争力。发展保税物流贸易，加快形成化工、矿石等大宗商品及民生消费品集散基地。加强服务外包载体建设，提速发展服务贸易。稳步扩大进口贸易，促进外贸平衡发展。完善国际贸易摩擦预警机制，增强防范应对能力。实行产业链招商，助推新兴产业规模化、集聚化。高度重视服务业利用外资，重点引进区域性总部和功能性机构。办好中国首届进口商品博览会。支持企业开展跨国经营，抓好千万美元以上的境外投资项目，努力将埃塞俄比亚东方工业园建设成为全国境外经贸合作样板区。探索苏州保税港区“一港三区”以及苏州综合保税区“一区四地”联动发展，争取设立海峡两岸（昆山）商贸合作先行先试区和中德（太仓）中小企业合作示范区。按照“在合作中有特色、在学习中有发展、在借鉴中有创新”的要求，推进苏州工业园区建设，深化中新友好合作。充分发挥外事、侨务、对台工作的重要作用，广泛深入开展对外交流。争取缔结1～2个与我市规模匹配、地位相当、优势互补的国际友城。主动融入全省沿海开发，加强南北挂钩合作，加快各类共建园区开发建设进度。做好对口援建工作，全面落实新疆霍尔果斯特殊经济开发区发展规划和产业规划。

　　四、着力强化城乡一体

　　整体推进城乡一体化发展综合配套改革，完善城乡一体化政策制度框架。统筹城乡基础设施建设和社会事业发展，促进共建共享共用。加大财政支农力度，落实强农惠农政策。进一步推动工业企业向园区集中、农业用地向规模经营集中、农民居住向新型社区集中，优化城乡资源配置。鼓励农户将集体资产所有权、土地承包经营权、宅基地及住房置换成股份合作社股权、城镇社会保障和城镇住房，加快农民身份转换。支持股份合作制经济转型升级，推动资源资产化、资产资本化、资本股份化和股份市场化，增强农村经济发展活力。加强集体资产管理，壮大农村集体经济。健全农民增收长效机制和利益保障机制，合理分配合作经济组织收益，让农民更多地分享土地增值收益。改善农村金融服务，用好城乡一体化投资基金。坚持农业生产、生活、生态、生物功能定位，加快农业现代化步伐。优化产业空间布局，注重产业融合发展，推进农业科技进步，提高农业综合效益。以园区化、农场化、合作化为导向，改造农业基础设施，转变农业生产方式，发展高效设施农业，抓好高标准示范片和万顷良田建设，建成现代农业园区面积6.7千公顷。严格保护基本农田，规范开展城乡建设用地增减挂钩工作。认真落实全国水利现代化试点市的各项任务，增强流域性水利工程的防洪保安能力和区域骨干河道的引排能力，推进农田水利和圩区堤防达标建设。提高村庄环境整治水平，严格防控农业面源污染，建立健全村庄设施维护、河道管护、绿化养护、垃圾收运、公厕保洁等队伍和长效管理制度，强化地方特色和乡村风貌塑造，努力实现环境优良、生态宜居。

　　五、着力推进城市建设

　　围绕中心城市“一核四城”的总体框架，高水准规划建设滨湖新城和高铁新城。优化平江、沧浪、金阊3个新城的功能及业态布局。在充分做好前期准备工作的基础上，争取沪通、通苏嘉、沿江等城际铁路尽早获准开工。建成苏州火车站南站房和南广场配套工程。建设常嘉高速公路、太仓疏港高速公路、张家港疏港高速公路、312国道苏州改线段，高标准完成苏虞张公路北段快速化改造。实施中环快速路以及东环、南环快速路延伸工程，力争尽早开工建设西环快速路北延工程，新建改建一批城市道路。确保苏州轨道交通1号线投入运营，2号线实现洞通，抓紧开工2号线延伸线和4号线。认真做好昆山花桥轨道交通与上海11号线的对接工作。结合轨道交通建设，科学高效开发利用地下空间资源。推进苏州港码头泊位建设，完成货物吞吐量4亿吨、集装箱吞吐量500万标箱。开展苏南运河、杨林塘等重要航道整治，续建张家港复线船闸。加大城乡电网新建改建力度，建成投运正负800千伏同里换流站。建设“城市光网”和“无线城市”，发展新一代移动通信，加快信息化进程，打造“智慧苏州”。加强人民防空工作，新增人防工程72万平方米。重视防治地质灾害，新建一批地震应急避难场所。实施气象监测和公共服务数字化工程，增强气象预测预报能力。整治规范城市交通秩序，科学规划和建设停车场，提高智能化管理水平，努力缓解交通拥挤状况。健全“数字城管”体系，推行市容环境精细化管理，强化路段、片长负责制，严格管理工程车、渣土车，优化美化城市形象。开展生活垃圾分类投放、收集试点工作，建成餐厨垃圾收集处置二期和七子山垃圾填埋场二期工程，建设生活垃圾焚烧发电三期工程和建筑垃圾处置场。

　　六、着力打造文化强市

　　牢牢把握先进文化的前进方向，推动社会主义文化大发展大繁荣。加强社会主义核心价值体系建设，广泛开展以讲文明、树新风为主题的公民道德实践活动，落实政务效能提升、文明素养培育、公共环境优化、公共事业普惠、交通文明共铸、城乡文明一体化推进和文明建设示范七大行动，完善社会志愿服务体系和激励机制，深化群众性精神文明创建工作，巩固提升全国文明城市建设水平，构筑全市人民共有的精神家园。重视未成年人心理健康教育，优化未成年人成长环境。坚持政府主导，按照公益性、基本性、均等性、便利性的要求，完善文化基础设施和公共文化服务网络，推行基层公共文化设施标准化，丰富群众文化生活，扩大文化惠民覆盖面，确保国家公共文化服务体系示范区创建达标。加强文化遗产保护，开展国家世界文化遗产监测试点，启动建设中国非物质文化遗产展示交流中心，探索建立非物质文化遗产项目生态保护示范区；结合中国大运河申报世界文化遗产，做好苏州古城相关点段申遗工作。大力实施虎丘地区综合改造、桃花坞历史文化片区综合整治保护利用、古城墙保护性修复和古建老宅保护修缮等工程。普及吴文化知识，精心编纂《苏州市志》和《苏州通史》。加快建设市档案馆新馆。新建苏州中国昆曲剧院。努力推出更多思想性艺术性观赏性相统一、群众喜闻乐见的优秀文艺作品。发展健康向上的网络文化和现代传播体系。办好第五届中国昆剧艺术节、第五届中国苏州评弹艺术节、第四届中国非物质文化遗产保护·苏州论坛、中国长江文化艺术节等文化活动。繁荣发展哲学社会科学，更好地服务经济社会发展。加强科普宣传，弘扬科学精神。深入实施文化产业跨越提升计划，推进文化科技创新，继续建设中国工艺文化城、中国光华（苏州）文化创意产业园、阳澄湖数字文化创意产业园、新闻出版大厦、现代传媒广场等重大项目，办好首届中国（苏州）文化创意设计产业交易博览会。创新文化管理体制，推进经营性文化单位改革，培育合格市场主体。构建统一开放竞争有序的现代文化市场体系，促进文化产品和要素合理流动。落实文化经济政策，加大财政对文化建设的投入力度。推动苏州文化“走出去”，广泛进行对外文化交流与传播。壮大基层文化工作者队伍，注重高层次高素质文化人才的培养与引进，努力造就一批文化名家大家。

　　七、着力加强生态建设

　　切实保护环境，狠抓节能减排，建设生态文明。加强集中式饮用水源地水质监测和污染风险防控，控制蓝藻、水草等生物灾害，确保饮用水安全。全面完成太湖流域水污染治理年度任务，继续推进东太湖、七浦塘综合整治和走马塘延伸拓浚等工程，加大城乡黑臭河道整治力度。加快污水处理厂和配套截污管网建设，提高生活污水处理率。开展更高水平的大气环境监测研究，进一步治理机动车尾气、工业废气和扬尘污染，努力减少灰霾天气。控制噪声污染，开展噪声达标区建设，重点查处夜间施工噪声扰民行为。有效防治化工、印染、电镀等工业污染，综合治理重金属污染，完成铅蓄电池行业整治任务。严格管理化工园区，完善环境风险源数据库，实行重大环境风险源动态监控。加强节能评估审查，从源头上控制能耗过快增长。推行合同能源管理，建立市场化节能服务机制。深化“能效之星”创建活动，提高企业节能降耗水平。普及节能知识和技术，倡导绿色低碳生活方式。盘活存量土地，强化节约集约用地考核，落实土地执法监管共同责任制。推广使用建筑节能新材料、新技术，加快城乡建设领域节能减排步伐。构建再生资源回收利用体系和循环产业链。巩固生态市建设成果，确保我市通过国家环保模范城市复核验收，力争吴江市和苏州市区进入全国生态文明建设试点地区行列。实施运河风光带、胥江河两岸景观等重大绿化工程，建成石湖滨湖景区，市区新增绿地450万平方米。推进农村绿化美化，开展生态公益林认定工作，构建河湖林网、绿色通道和生态片林，农村新增林地绿地4667公顷，陆地森林覆盖率达到26%。实施丘陵山地复绿和林相改造，基本完成山体宕口整治任务，加强公墓管理，保护山体植被。高度重视湿地保护与管理，建设环太湖湿地保护区、北部沿江湿地保护区和中南部湖荡湿地保护区。

　　八、着力发展社会事业

　　适应人民群众需求增长，推动社会事业全面进步。高水准推进教育现代化，优化配置城乡幼儿园、中小学，新建幼儿园、小学、初中全部达到市高水平教育现代化学校标准。积极创建省学前教育改革发展示范区，认真开展国家义务教育优质均衡改革发展试点、高等职业教育国家综合改革试点等工作。建设市第十中学金阊新城校区，启动迁建市幼儿师范学校和第五中学，改扩建市老年大学、高等职业技术学校、旅游与财经高等职业技术学校，完成苏州中学校园整体改造和中小学校舍安全工程。支持在苏高校加强内涵建设，促进高等教育优质创新发展。拓展优质医疗资源，优化医疗机构设置，加快推进医院在建工程，适时启动市公共医疗中心项目，开工建设金阊新城医院。健全基层公共卫生服务体系，完善基本药物目录，促进医药卫生城乡一体化。推动医院与基层医疗卫生机构之间的双向转诊，开展电话预约挂号便民服务。实施全民健康促进“百千万”工程，新增一批健康镇村、健康社区、健康单位。加强职业健康监护，防治职业病。围绕基本实现体育现代化的要求，贯彻落实全民健身条例，积极构建“十分钟体育健身圈”；办好市第13届体育运动会，建成市网球中心。加强人口调控，注重家庭发展，深化城乡一体化人口和计划生育综合改革。开展孕前优生检查、青少年健康人格塑造、流动人口均等服务等工作。保障少数民族合法权益，依法管理宗教事务。实施妇女儿童“十二五”规划，维护妇女儿童权益，提高妇女儿童事业发展水平。

　　九、着力改善人民生活

　　持续增加民生事业投入，办好各项惠民利民实事，让人民群众共享改革发展成果。认真落实城乡居民收入6年倍增计划，建立与经济增长相适应的居民收入正常增长机制。加大收入分配调节力度，努力提高中低收入者的收入水平，逐步缩小收入分配差距。拓宽公益性岗位范围，广泛实行就业再就业援助。开展大学生就业促进行动，组织提供高校毕业生就业岗位10万个。继续创建充分就业社区、充分就业村，对城镇零就业家庭实行动态清零。发挥职业教育优势，加强职业技能培训，增强劳动者就业能力，免费培训城乡劳动者30万人。认真贯彻各项鼓励创业的政策和帮扶引领计划，争创国家创业型城市。巩固城镇职工社会保险覆盖面和社会保险基金征缴率。改革失业保险制度，推进医疗保险、生育保险市级统筹，实现养老保险和居民医疗保险城乡并轨。重视扶贫开发，加大对经济薄弱地区的帮扶力度，增强其自我发展能力。稳步提高城乡居民最低生活保障水平。健全社会救助制度，重点解决因病致贫家庭的生活困难，适度扩大临时救助范围。鼓励慈善募捐，发展红十字事业，加强评估监督。建成市社会福利总院一期工程。适时提高最低工资标准，依法开展工资集体协商。发展社会养老事业，新增养老床位6000张、日间照料中心40家、助餐点100个，改进居家养老服务。加强残疾预防和早期筛查，开展居家养残服务，增设一批助残点，提升残疾人康复服务水平。加快建设保障性住房，努力让符合标准的家庭享受住房保障。扩大住房公积金保障覆盖面，为基本住房消费提供更多支持。大力实施南环新村危旧房解危改造工程，启动第二批城区危旧房解危改造。继续推进城区居民家庭“改厕”，综合整治老住宅小区和零星居民楼，解危修缮直管公房，改造城中村（无地队）。加强校园安全工作，为学生接送车辆全部安装GPS动态安全监管系统。坚持公交优先，建设公交专用道和路口优先道，优化公交线网和站点布局，提供更加便捷的公交服务；更新公交车辆，市区新增公交车500辆，推行公交智能化、信息化管理，推广出租车电话召车。倡导文明经营、诚信经营，改善消费环境。抓好“菜篮子”工程，新建改建一批城乡农贸市场，增加农产品平价直销网点，保障农产品供应充足、价格基本平稳。稳定粮食生产，增加粮食储备，改扩建白洋湾金仓粮食中转库，建设一批中心粮库和骨干粮库。建立食品安全监测预警体系和农产品质量安全监测体系，严防食品安全事故发生。开展药品安全责任体系评价试点，力争各县级市全部成为省级以上药品安全示范县（市）。强化市场价格监管，维护市场价格秩序，及时发布重要商品价格信息，努力控制消费价格总水平过快上涨。

　　十、着力促进社会和谐

　　加强民主法治建设，维护社会和谐稳定。坚持依法治市，弘扬社会主义法治精神，提高法治化管理水平。深入推进基层民主法治工作，积极创建全国、全省民主法治示范村和示范社区。创新社区管理方式，增强社区服务功能，促进社区自治，创建和谐社区、文明社区。抓好“六五”普法开局工作，为争创全国“六五”普法先进单位打下坚实基础。办好法律援助窗口，提升便民法律服务实效。把社会建设摆上更加突出的位置，创新和完善社会管理工作体系，努力形成科学有效的群众利益协调机制、劳动关系协调机制、诉求表达机制、矛盾调处机制、权益保障机制和社会稳定风险评估机制。注重建立高素质的社会工作者队伍，推进社会服务专业化。深化平安苏州建设，加强社会管理综合治理，排查整治治安突出问题，严厉打击违法犯罪行为和境内外敌对势力的渗透破坏活动。做好预防青少年违法犯罪、刑释解教人员安置帮教和社区矫正、肇事肇祸精神病人服务管理等工作。坚持以人民调解为基础，完善社会矛盾纠纷大调解机制，层层落实信访工作责任，努力在第一时间、第一地点预防和化解社会矛盾。健全公共安全体系和应急管理体制机制，增强处置突发事件的能力。一丝不苟地履行安全生产监管责任，推进高危行业、公共领域安全标准化和安全监管信息化建设，开展重点领域专项整治和重大隐患挂牌整改，严防重特大事故发生。强化流动人口服务和管理，积极实施居住证制度，建立流动人口流入地和流出地协作机制。坚持建设与管理并重、发展与管理同步，加快形成法律规范、行政监管、行业自律、技术保障、公众监督、社会教育相结合的信息网络管理体系。支持驻苏解放军和武警部队建设，做好国防动员和民兵预备役工作。拓展深化双拥创建，巩固和发展新型军政军民关系。

　　各位代表，苏州正处在率先基本实现现代化的关键阶段，使命和目标激励着我们，责任和重托鞭策着我们，困难和挑战考验着我们。完成今年的各项目标任务、向全市人民交上一份满意的答卷，必须认真贯彻落实以人为本、执政为民的要求，加强和改进政府自身建设，更好地履行经济调节、市场监管、社会管理和公共服务职能，努力提高工作水平。实施法治政府建设五年规划，切实按照法定权限和法定程序行使权力，自觉运用法律手段调节、管理经济和社会事务。高质量做好政府立法工作，开展规章实施后评估，严格实行规范性文件备案审查。建设依法行政示范点，完善行政执法体制，规范行政处罚行为。依法开展行政复议和应诉工作，全面推行行政调解。进一步抓好机关作风效能建设，加强对领导干部、机关部门和直接服务企业、服务群众的重点处室的作风考评，积极创建机关优质服务品牌。严格公务员教育和管理，提高责任感和执行力。增强行政服务中心功能，继续推进集中审批、联合审批和网上审批，更好地方便企业和群众。加强便民服务中心能力建设，优化政风行风热线服务。继续深入开展民主评议政风行风活动，坚决纠正损害群众利益的不正之风。深化政务公开内容，丰富政务公开形式，坚决做到行政权力公开透明运行，保障人民群众的知情权、参与权和监督权。各级政府要自觉接受人民代表大会及其常务委员会的法律监督、工作监督，接受人民政协的民主监督，接受司法机关的监督，接受新闻舆论和社会公众的监督，认真实施人大议案，办理代表建议和政协提案。健全惩治和预防腐败体系，强化从源头上防治腐败。大力整治工程建设领域的突出问题，严厉打击违规项目审批、规划调整以及规避招标、虚假招标、转包、违法分包等行为。发扬艰苦奋斗的优良传统，坚持勤俭节约，杜绝铺张浪费。严格控制公车购置使用，完善因公出国（境）管理机制，规范会议、公务接待以及各类庆典、研讨会、论坛等活动，降低行政成本。加强政府性资金绩效审计，抓好领导干部经济责任审计，认真开展内部审计，积极推进审计结果公开工作，强化审计整改，促进审计成果综合利用。

　　各位代表，时代赋予重任，人民寄予厚望。让我们紧密团结在以胡锦涛同志为总书记的党中央周围，在中共苏州市委的坚强领导下，齐心协力，奋发进取，扎实工作，为加快“三区三城”建设、率先基本实现现代化而努力奋斗，以优异成绩迎接党的十八大胜利召开！
